# Majocchi Granuloma Superinfected With Herpes Zoster

**DOI:** 10.7759/cureus.62075

**Published:** 2024-06-10

**Authors:** Donald Lei, Elif Karatas, Daniel Antohi, Benedict Wu

**Affiliations:** 1 Dermatology, Albert Einstein College of Medicine, Bronx, USA; 2 Dermatology, Beth Israel Deaconess Medical Center, Harvard Medical School, Boston, USA

**Keywords:** varicella zoster reactivation disseminated herpes zoster, fungal infection, superinfection, herpes zoster virus, majocchi’s granuloma

## Abstract

Herpes zoster (HZ) infection is caused by the reactivation of the varicella-zoster virus (VZV) and has very rarely been reported at the site of a superficial fungal infection. Also, HZ occurring at the site of a deep fungal infection has not been reported in the literature. We discuss a unique case of a 45-year-old male patient presenting with a Majocchi granuloma (MG) superinfected with disseminated HZ.

## Introduction

Herpes zoster (HZ) results from the reactivation of the varicella-zoster virus (VZV) and often presents as a painful vesicular eruption along a dermatome. HZ can be superinfected by Staphylococcus and Streptococcus species, even though it has rarely been reported in conjunction with fungal infections [1]. To our knowledge, less than 10 cases of HZ occurring at the site of a fungal infection have been reported in the literature - all of them superficial fungal infections [2-4]. In contrast, we describe a unique case of Majocchi granuloma (MG) - a rare fungal skin infection characterized by dermal and follicular invasion of organisms typically limited to the superficial epidermis that is often treated with oral, rather than topical antifungals - occurring in the setting of disseminated HZ [5].

## Case presentation

**Figure 1:**
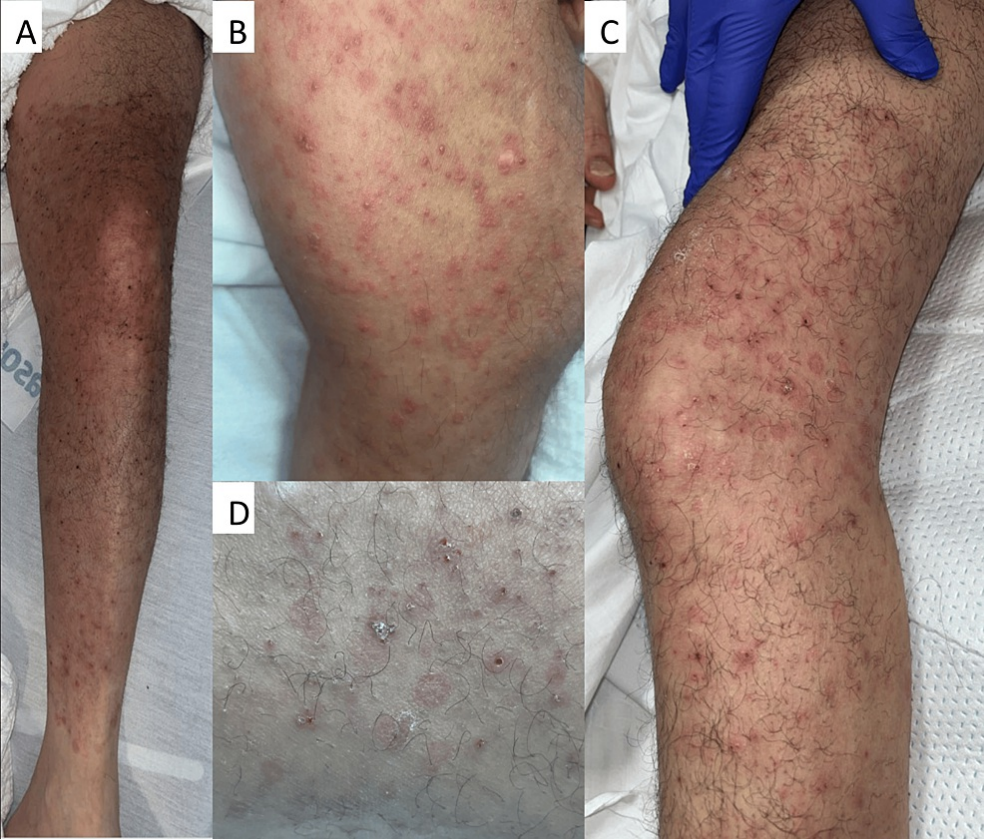
Patient images - left lower extremity A and B: the initial presentation; erythematous papules and vesicles on a well-demarcated plaque extending from the proximal left thigh to the ankle. C and D: after one day of IV acyclovir; progression to scaly, annular papules and plaques and crusted red perifollicular papules

A 45-year-old male with a past medical history significant for myasthenia gravis was admitted for a lumbar compression fracture, and his hospital course was complicated by postoperative acute hypoxemic respiratory failure requiring intubation. Dermatology was consulted for evaluation of an acute on chronic pruritic skin eruption affecting the back, buttocks, and lower extremities. The rash was reported to have been ongoing for over a year, affecting the back and buttocks, and spreading to the left lower extremity over the past few days. The initial physical exam was notable for erythematous papules and vesicles distributed across the back, buttocks, and left lower extremity. Notably, starting at the proximal thigh and ending at the ankle of the left lower extremity, there were many perifollicular papules and crusted erosions within an annular plaque (Figure 1A).

A viral PCR swab from a vesicle was positive for VZV, and the patient was initiated on IV acyclovir. After one day of acyclovir, the erythematous scaly annular, perifollicular papules became more prominent on the left lower extremity (Figure 1B). A punch biopsy from one of the perifollicular papules with surrounding scale showed intraepidermal acantholysis and suppurative granulomatous dermatitis, consistent with a viral vesicular dermatitis. A PAS-D stain highlighted fungal hyphae in the stratum corneum and budding yeast in the suppurative dermal inflammation, consistent with a diagnosis of both HZ and MG. The patient was then started on twice-daily topical ketoconazole cream and oral itraconazole 200 mg twice daily for one week, repeated monthly for three months.

## Discussion

HZ is caused by the reactivation of VZV infection, thought to be due to the immune system’s inability to control viral replication in its dormant state. Thus, HZ is strongly associated with states of impaired immunity, such as advanced age, stress, malignancy, or immunocompromise. A large nationwide inpatient study showed a positive association between hospitalized patients with chronic inflammatory skin disease and the incidence of HZ, further supporting this link between HZ and the immune system [6]. Thus it is not surprising that bacterial superinfections with Staphylococcal or Streptococcal species are some of the most commonly reported complications of HZ [7].

Interestingly, while bacterial superinfections are common, fungal superinfections are very rarely described in the literature. Fewer than 10 cases of concomitant fungal infection and herpes zoster are described in the literature - all of them involving superficial dermatophyte infections [2,3,8]. We highlight this point to illustrate the rarity and novelty of the case presented. In our patient, as confirmed by positive PAS-D staining of fungal hyphae in the dermis, a Majocchi granuloma was diagnosed after HZ treatment was initiated. Majocchi granuloma is a rare entity itself, defined by follicular and dermal invasion by fungal organisms that typically only affect the superficial epidermis. This depth of the infection means that MG often requires oral antifungal treatment for weeks, rather than topical azole treatment which is often sufficient for more superficial fungal infections like tinea [9]. 

In addition, this case is interesting from both morphologic and pathophysiologic perspectives. The patient’s initial lesions - an erythematous popular and vesicular eruption - were a classic presentation of HZ. However, clearance following antiviral therapy gradually unmasked scaly, annular papules and plaques in a follicular distribution which correlates to the dermal and follicular involvement seen in Majocchi granuloma. Furthermore, well-demarcated and scaly proximal and distal margins of the lesion were observed.

One hypothesis for the overlapping lesions seen in this patient is that the patient’s fungal infection precipitated a localized area of cutaneous immune dysregulation. This phenomenon, first described by Ruocco and hence known as Ruocco’s immunocompromised cutaneous district (ICD), refers to an initial dermatosis or cutaneous infection that causes increased susceptibility to future dermatoses such as HZ [10]. Though the exact mechanisms are not understood, it has been postulated that persistent fungal antigen expression can lead to local T-cell exhaustion, predisposing individuals to HZ infection [2].

## Conclusions

We believe this report will encourage clinicians to consider fungal and bacterial etiologies as important causes of superinfection or co-infection with HZ. We also hope this case helps illustrate the unique morphologic clues suggestive of MG that may only manifest after an initial HZ infection is treated, which highlights the importance of interval monitoring of HZ lesions.
